# Secondary hypertension: An update on the diagnosis and localisation of a pheochromocytoma or paraganglioma

**DOI:** 10.4102/safp.v63i1.5277

**Published:** 2021-07-14

**Authors:** Nida Siddiqui, Reyna Daya, Faheem Seedat, Saajidah Bulbulia, Zaheer Bayat

**Affiliations:** 1 Department of Internal Medicine, Division of Endocrinology, Diabetes and Metabolism, Faculty of Health Sciences, Helen Joseph Hospital, Johannesburg, South Africa

**Keywords:** secondary hypertension, endocrine, pheochromocytoma, paraganglioma, screening, normetanephrines, metanephrines, succinate dehydrogenase

## Abstract

Most cases of hypertension are because of essential hypertension, however 5% – 15% of cases can be a result of a secondary cause. In this article, we focus on the endocrine causes of secondary hypertension with a particular focus on pheochromocytomas (PCCs) and paragangliomas (PGLs). Around 15 endocrine disorders can initially present with hypertension. Amongst those PCCs and PGLs are rare but potentially life-threatening causes. An early diagnosis and timely referral can be life-saving. Herein, we present an approach for screening and diagnosis of these patients and focus on the importance of genetic testing.

## Introduction

An estimated 1.39 billion people in the world have hypertension (HT), with approximately 9 million related annual deaths worldwide.^1,2^ In South Africa, one in four adults have HT; however, a prevalence of up to 60% has been reported.^3^ Most of these patients have no clear aetiology and are classified as having essential or primary HT. However, 5% – 15% have secondary HT, which is defined as HT because of an underlying cause.^4,5^

Hypertension is a serious medical condition requiring timely and sustained treatment. The economic cost of HT includes both direct costs, such as treatment of the disease and its associated chronic conditions and indirect costs such as early mortality and loss of productivity. Quantifying the economic costs of HT is particularly challenging as it is an independent risk factor for many chronic conditions such as cardiovascular, cerebrovascular and chronic kidney disease.^6^

Hypertension is defined as a consistently high systolic blood pressure (BP) of more than 140 mm of mercury (mmHg) or diastolic BP of more than 90 mmHg.^5^ The diagnosis of HT should be confirmed at a second visit, usually 1–4 weeks after the initial elevated BP reading. If white-coat HT is suspected, home BP monitoring should be considered, using the average BP measured over 5–7 days.^3,7^

As a result of the prevalence of secondary HT being relatively low, routine testing for a secondary cause in all cases of HT is neither cost effective nor practical. Moreover, it can be challenging for clinicians to know when and how to perform case-detection testing for the various disorders, which may present with HT. Here, we discuss an approach to secondary HT and review different causes of endocrine and non-endocrine HT with a focus on the pheochromocytoma and paraganglioma (PPGL) syndromes.

## Secondary hypertension

Secondary HT is an elevated BP because of an identifiable, potentially correctable cause.^8^ The following are clinical clues that could lead to the diagnosis of secondary HT.^3,8,9,10^

- Age of onset of HT before puberty
- Age less than 30 years in patients with no family history of HT or other risk factors
- Labile BP or an acute rise in BP in a patient with previously stable BP readings
- Resistant HT, for example, persistently elevated BP greater than 140/90 millimetre of mercury (mmHg) despite using adequate doses of three antihypertensive agents from different classes, one of which is a diuretic
- Hypertension associated with electrolyte disorders such as hypokalemia or metabolic alkalosis
- Malignant HT, for example, patients with severe HT (BP greater than 180/110 mmHg) with end organ damage (left ventricular hypertrophy, hypertensive retinopathy, flash pulmonary edema, acute kidney injury or neurological manifestations)
- Unexplained or acute persistent elevation of serum creatinine of at least 30% after administration of angiotensin-converting enzyme (ACE) inhibitor, angiotensin receptor blocker (ARB) or renin inhibitor
- Moderate to severe HT in a patient with diffuse atherosclerosis, a unilateral small kidney or asymmetry in kidney size of more than 1.5 cm that cannot be explained by another reason.

The causes of secondary HT can be broadly subdivided into endocrine, renovascular, renal parenchymal, vascular and other (Table 1).^4,9,10,11^

**TABLE 1:** Endocrine and other causes of secondary hypertension.

Causes	Examples
***Endocrine*^4^**	**Pituitary dependent causes** Acromegaly Cushing’s disease **Thyroid-dependent causes** Hypothyroidism Hyperthyroidism **Parathyroid-dependent causes** Hyperparathyroidism **Adrenal-dependent causes** Pheochromocytoma Sympathetic paraganglioma Primary aldosteronism Hyperdeoxycorticosteronism ■ Congenital adrenal hyperplasia: (1) 11 b-hydroxylase deficiency, (2) 17 a-hydroxylase deficiency ■ Deoxycorticosterone-producing tumours ■ Primary cortisol resistance Cushing’s syndrome **Apparent mineralocorticoid excess/11 b-hydroxysteroid dehydrogenase deficiency** Genetic Acquired ■ Licorice ingestion ■ Cushing’s syndrome
***Renal*^10^**	**Renal parenchymal** Chronic kidney disease Polycystic kidney disease **Renal vascular** Renal artery stenosis Fibromuscular dysplasia Vasculitis, for example, Takayasu’s arteritis Extrinsic compression of renal artery Renal artery dissection or infarction
***Vascular*^10^**	**Coarctation of the aorta**
***Other*^11^**	**Drugs** Non-steroidal anti-inflammatory drugs Oral contraceptive pills, androgens Illicit drugs such as amphetamine and cocaine Steroids, for example, prednisone Herbal pills, for example, ephedra, ginseng Psychiatric drugs, for example, tricyclic anti-depressants (TCAs), selective-serotonin reuptake inhibitors (SSRIs), monoamine oxidase inhibitors (MOIs), carbamazepine, clozapine Immunosuppressive agents, for example, cyclosporine, sirolimus, tacrolimus **Obstructive sleep apnea**

*Source*: Table adapted with permission from Young et al.^4^ and information from Charles et al.^11^

## Endocrine hypertension

Around 15 endocrine disorders may initially present with HT.^4^ Amongst these, primary hyperaldosteronism is the most common cause and therefore should be considered in patients as an underlying cause of secondary HT.^12^ Primary hyperaldosteronism is excess production of aldosterone, independent of the renin–angiotensin system and may be caused by an adrenal adenoma, unilateral or bilateral adrenal hyperplasia or adrenocortical carcinoma.^4^

Other causes of secondary HT such as PPGL syndromes albeit less prevalent in the general population, present with a higher prevalence in patients with HT. A missed diagnosis may have life-threatening consequences and hence even the slightest clinical suspicion should prompt biochemical testing for PPGL.^4^

## Pheochromocytoma and paraganglioma

Pheochromocytoma and paragangliomas are a collective term encompassing both pheochromocytoma (PCC) and paraganglioma (PGL). Paragangliomas are rare neuroendocrine chromaffin cell tumours with a combined annual incidence of 0.8 per 10 000 persons per year globally.^4,13^ As a result of limited healthcare resources, there is lack of data from the African population, especially from Southern Africa. A 30-year and a 14-year audit performed in two of the largest academic hospitals of South Africa showed that HT was the predominant clinical finding (100% and 85%, respectively) in patients who presented with PPGL syndromes.^14,15^

Pheochromocytoma and PGL are also known as the ‘great mimic’ because of the non-specific clinical presentation such as sweating, palpitations, headaches and anxiety, which may overlap with many other medical disorders.^16^

Pheochromocytomas arise from the adrenal medulla, whilst PGLs arise from the paravertebral ganglia of the sympathetic chain.^4,17,18^ Paragangliomas which arise from parasympathetic tissue in the head and neck are mostly silent (i.e. they do not secrete any catecholamines).^19^

The traditional rule of 10 suggested that 10% of PPGLs will be bilateral, extra-adrenal and metastatic. However, advances in the fields of genetics and diagnosis have challenged this rule.^17^ Prevalence of bilateral and extra-adrenal PPGL is now estimated to be 10% – 15%, whilst 30% of extra-adrenal PGLs are said to be metastatic. It is estimated that around 40% of PPGLs have a hereditary cause.^16,17^

**FIGURE 1:**
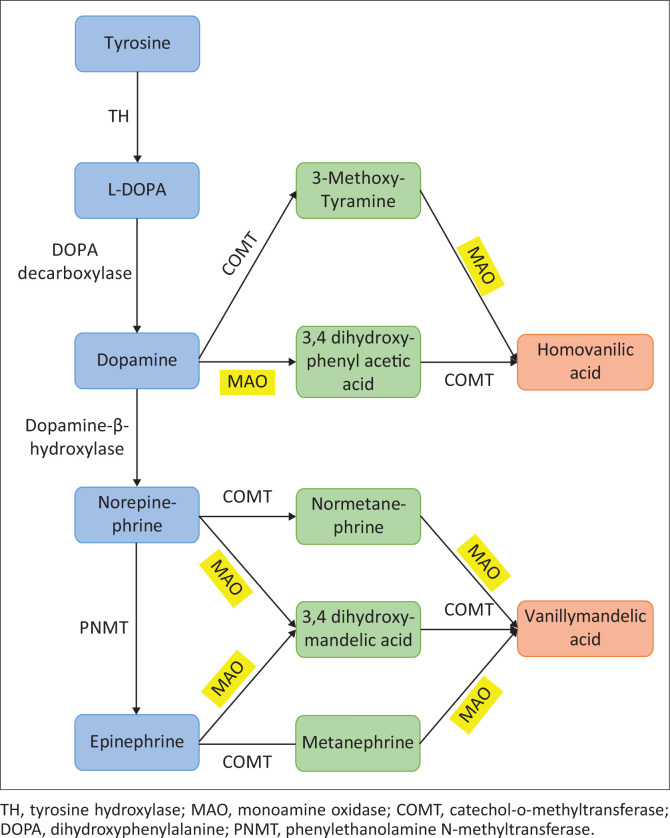
Metabolic pathway of catecholamines.

Pheochromocytoma and PGLs may present with at least four different biochemical phenotypes (adrenergic, noradrenergic, dopamine producing or silent) as described here (Figure 1).^18^

- Adrenergic tumours that are located in the adrenal medulla and produce epinephrine, norepinephrine and metanephrine (major metabolite of epinephrine).
- Noradrenergic tumours are located either in the adrenal medulla or extra-adrenal sites and produce norepinephrine.^4^ The lack of epinephrine secretion in extra-adrenal tumours is because of the absence of the enzyme phenylethanolamine-N-methyltransferase (PNMT), which is essential for converting norepinephrine to epinephrine.^20^
- Dopamine-producing tumours are associated with dopamine or its metabolite 3-methoxytyramine production. These tumours usually lack dopamine-b hydroxylase, which is responsible for converting dopamine to noradrenaline. They are mostly associated with an underlying succinate dehydrogenase (SDH) mutation.^18^
- Silent tumours are tumours that do not produce any catecholamines or their metabolites. They can present anywhere but are most common in the head and neck region (HNPGL). They are often diagnosed because of mass effects such as pain or cranial nerve compression.^21^

The biochemical phenotype is important for predicting the underlying germ-line mutation, for example, SDH and von-Hippel Lindau (VHL) mutations have tumours that usually produce norepinephrine, whereas multiple endocrine neoplasia type 2 (MEN2) and neurofibromatosis type 1 (NF1) are more likely to be associated with tumours producing epinephrine. Biochemical phenotype can also determine the clinical presentation of the tumour, for example, adrenergic tumours often present with paroxysms of symptoms whereas noradrenergic tumours usually present with sustained HT.^4^

## Screening and imaging for pheochromocytoma and paraganglioma

Clinicians should have a low index of suspicion when deciding which patients should have biochemical testing for PPGL. Screening should be considered in the following situations^4^:

- patients with HT who present with paroxysmal symptoms of sweating, headaches and palpitations
- resistant HT (as described earlier)
- incidental adrenal mass with or without HT
- family history of PPGL
- syndromic features indicating a PPGL-related hereditary syndrome
- previous diagnosis of PPGL (annual biochemical testing to detect recurrent disease)
- paradoxical BP response to anaesthesia or drugs, for example, beta-blockers
- paroxysmal HT.

Biochemical testing should include the measurement for plasma free or 24-h urinary fractioned metanephrines (sensitivity of 99% and 97%, respectively).^4^ Plasma metanephrines remain the gold standard^4^ and testing is currently available at a few private laboratories in South Africa. Plasma metanephrine testing is currently unavailable at the National Health Laboratory Service (NHLS). Diagnostic accuracy is not significantly different between plasma and urine metanephrines and normal values of these tests are often enough to exclude a PPGL.^4,17^

Excess secretion of metanephrines or normetanephrines, three to four times above the upper reference range are typically pathognomonic for PPGL.^4,17^ It is important to note that a mildly elevated fractioned metanephrine levels (plasma or urine) are not specific for the presence of PPGL, therefore testing should not be conducted in critically ill patients or conditions, which can increase the sympathetic activity or with concurrent use of certain drugs (Table 2).^8^

**TABLE 2:** Conditions or drugs that can lead to false-positive results.

Causes	Examples
Conditions which increase sympathetic activity	Heart failure Renal failure Hypoglycemia
Drugs	TCAs Monoamine oxidase inhibitors Antipsychotics SSRIs Levodopa Phenoxybenzamine Beta-blockers such as labetalol, sotalol Alpha-methyldopa Sulfasalazine

*Source*: Adapted with permission from Young WF Jr, Calhoun DA, Lenders JWM, Stowasser M, Textor SC. Screening for endocrine hypertension: An endocrine society scientific statement. Endocrine Rev. 2017;38(2):103–122. https://doi.org/10.1210/er.2017-00054

SSRI, selective-serotonin reuptake inhibitor; TCA, tricyclic anti-depressant.

Other available biochemical tests, such as urinary catecholamines (sensitivity of 86%), urinary vanillylmandelic acid (sensitivity of 64%) and plasma chromogranin A (sensitivity of 90%) have inferior diagnostic value as compared with urinary fractioned metanephrines.^4,17,22^

**FIGURE 2:**
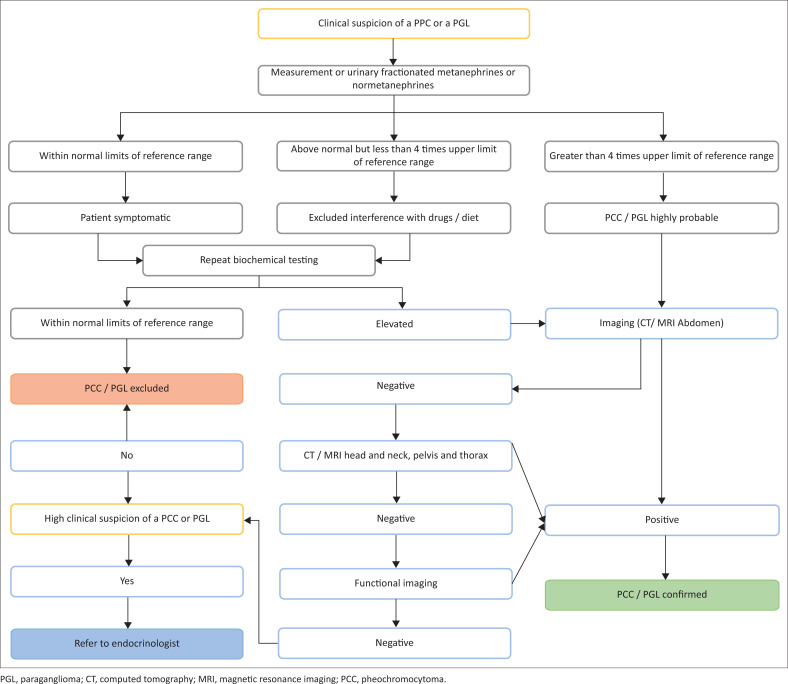
Approach to a patient with suspected pheochromocytoma and paraganglioma.

Once obvious causes of falsely elevated test results are ruled out (Table 2) and a biochemical diagnosis of PPGL has been made, clinicians should then proceed with medical imaging (Figure 2).^23^ The tumour can be localised with either contrast-enhanced computed tomography (CT) or magnetic resonance imaging (MRI).^16^ The 2014 Endocrine Society Guidelines suggest CT rather than MRI as the first-choice imaging modality.^24^ Clinicians should consider MRI in patients where there is a contraindication to contrast-enhanced CT scans such as an allergy to contrast medium, pregnancy or chronic kidney disease. Standard imaging should include the entire retroperitoneum as most extra-adrenal tumours are located in the retroperitoneum rather than in the pelvis or thorax. If an extra-adrenal tumour or tumour of more than 10 cm is detected, clinicians should aim to look for multifocal or metastatic disease.^16^

Functional imaging (scintigraphy) is effective in localising PPGLs and detecting multifocal or metastatic disease,^16^ and any of the following imaging techniques may be used; 123I-labelled metaiodobenzylguanidine (MIBG) or positron-emission tomography [PET]-CT with 68Ga-labelled 1,4,7,10 tetrazocyclododecane-1,4,7,10 tetra aceticacid-octreotate [DOTATATE] or 18F-labelled L-dihydroxyphenylalanine [L-DOPA].

Once biochemically confirmed, ideally all patients warrant referral to an endocrinologist or endocrine or a tertiary care centre for further investigation and management.^4^

Regardless of symptoms or biochemistry, we recommend that the following patients should also be referred to an endocrinologist or endocrine tertiary care centre:

- family history of PPGL
- syndromic features of a hereditary syndrome with known association with PPGL such as MEN2, NF-1 and VHL
- previous diagnosis of PCC or PGL (annual biochemical testing to detect recurrent disease)
- confirmed PPGL.

## Genetics of pheochromocytoma and paraganglioma

There are currently more than 20 susceptibility genes identified that cause PPGL.^16^ Some of the well-known associations include MEN2, VHL and NF1. Of late, mutations of the *SDH* gene have been identified. These mutations are responsible for PGL syndromes 1–5, caused by five distinct mutations of the *SDH* gene. In addition, novel mutations in the genes encoding transmembrane protein 127 (*TMEM127*) and MYC-associated factor X (*MAX*) have been described to cause hereditary PPGL syndromes.^16^

In an ideal situation all patients with PPGL should be referred for genetic screening and if a germline mutation is found, then all first-degree relatives should be offered mutation-specific gene testing. However, in a resource limiting setting, we recommend that genetic testing be directed to patients who present with any of the following characteristics:

- bilateral adrenal PCC
- unilateral adrenal PCC in a patient under 60 years of age
- multifocal disease or malignant disease
- patient with a PGL regardless of age
- patients with a clinical syndrome associated with PPGL and patients with a family history of PPGL
- clinical findings suggestive of a related syndromic disorder.

The NHLS is currently working on offering next generation sequencing (NGS) to test for germline mutations associated with PPGL. Considering that more than 40% of patients who present with PPGLs have a hereditary cause, the availability of genetic testing will greatly improve our chances to detect and offer genetic counselling and treatment to affected patients and their families.

## Conclusion

Hypertension is one of the leading causes of morbidity and mortality in South Africa.^25^ Rare causes of secondary HT such as PPGL syndromes are often missed, therefore a high index of suspicion is required by clinicians.

The majority of patients with PPGL present with either sustained or paroxysmal HT whilst a minority may be normotensive. Symptoms because of catecholamine excess can mimic over 30 medical disorders and thus can be confusing for the clinician to know when and how to perform case-detection studies for that rare patient who presents with an underlying PPGL. Over the years, clinical presentation of these syndromes has evolved from the classic triad of palpitations, headaches and sweating to incidental discovery of an adrenal mass (incidentaloma).

Early diagnosis thus depends on keen clinicians who recognise the signs and symptoms of catecholamine excess. In a resource limited setting where not all investigations are always available, rapid recognition, biochemical screening and timely referral to a tertiary care centre are of utmost importance, because missing the diagnosis may have fatal consequences.

The availability of genetic testing in the near future will greatly improve our chances to detect and offer genetic counselling and treatment to affected patients and their families.
